# Roles for the methyltransferase SETD8 in DNA damage repair

**DOI:** 10.1186/s13148-022-01251-5

**Published:** 2022-03-04

**Authors:** Libo Xu, Ling Zhang, Jicheng Sun, Xindan Hu, Dhan V. Kalvakolanu, Hui Ren, Baofeng Guo

**Affiliations:** 1 Department of Surgery, China-Japan Union Hospital of Jilin University, Changchun, People’s Republic of China; 2 Key Laboratory of Pathobiology, Ministry of Education, and Department of Pathophysiology, College of Basic Medical Sciences, Jilin University, Changchun, People’s Republic of China; 3 Greenebaum NCI Comprehensive Cancer Center, Department of Microbiology and Immunology, University of Maryland School Medicine, Baltimore, MD USA

**Keywords:** SETD8/PR-Set7/Set8/KMT5a, H4K20, Histone methylation, 53BP1, BRCA1, DNA damage repair

## Abstract

Epigenetic posttranslational modifications are critical for fine-tuning gene expression in various biological processes. SETD8 is so far the only known lysyl methyltransferase in mammalian cells to produce mono-methylation of histone H4 at lysine 20 (H4K20me1), a prerequisite for di- and tri-methylation. Importantly, SETD8 is related to a number of cellular activities, impinging upon tissue development, senescence and tumorigenesis. The double-strand breaks (DSBs) are cytotoxic DNA damages with deleterious consequences, such as genomic instability and cancer origin, if unrepaired. The homology-directed repair and canonical nonhomologous end-joining are two most prominent DSB repair pathways evolved to eliminate such aberrations. Emerging evidence implies that SETD8 and its corresponding H4K20 methylation are relevant to establishment of DSB repair pathway choice. Understanding how SETD8 functions in DSB repair pathway choice will shed light on the molecular basis of SETD8-deficiency related disorders and will be valuable for the development of new treatments. In this review, we discuss the progress made to date in roles for the lysine mono-methyltransferase SETD8 in DNA damage repair and its therapeutic relevance, in particular illuminating its involvement in establishment of DSB repair pathway choice, which is crucial for the timely elimination of DSBs.

## Background

Posttranslational modifications of histones on their N-terminal tails, comprising acetylation, methylation, phosphorylation, ubiquitination, and ADP-ribosylation are essential for regulating several biological processes [1, 2]. Among these, histone methylations predominantly occur on the lysine and arginine residues, playing a critical role in modulating chromatin dynamics with diverse epigenetic functions [3]. So far, lysine methylation of histones is best understood [3]. Methylation increases the hydrophobicity and diminishes the basic nature of the lysine, which allows other proteins to recognize the methylated lysine [4]. Methylation of lysine on histones at specific sites exerts distinct effects, e.g., methylation of H3K27 and H3K9 is associated with transcriptional silencing, whereas that of H3K4 and H3K36 are linked to gene activation [3, 5]. The complexity of histone lysine methylation has a significant impact on cell behavior, making it one of the most interesting posttranslational modifications. Histone H4 methylation is an intriguing histone modification with the majority present on its unique N-terminal tail of lysine 20 (H4K20me) [1]. Three methylation states of H4K20, including H4K20me1, H4K20me2 and H4K20me3, catalyzed by different methyltransferases, have been identified [6–8]. SETD8 is so far known as a sole mono-methyltransferase that catalyzes H4K20me1 in metazoans, whereas other methyltransferases, such as SUV4-20h1/h2, contribute to the transition from H4K20me1 to H4K20me2/3 [3, 6]. Although separate enzymes control each level of methylation, H4K20me2/3 is dependent on H4K20me1 [6–8]. Therefore, it is not surprising that the consequence of losing catalytic activity of SETD8 is more severe than that of di- or tri-methyltransferases as it precludes any levels of methylation on H4K20.

Many eukaryotic cells encounter a variety of threats during their life span with a high possibility to generate DNA damage, some of which bare serious consequences [9, 10]. The DNA double-strand breaks (DSBs) represent perhaps the most toxic DNA damage that can drive oncogenic mutations and chromosomal rearrangements if unrepaired [9, 11, 12]. Several dedicated DNA repair mechanisms have thus evolved to eliminate such aberrations to maintain genome integrity [13]. The homology-directed repair (HDR) and canonical nonhomologous end-joining (c-NHEJ) are two most prominent DSB repair strategies [9–12]. HDR re-ligates using error-free homologous DNA sequences as a template primarily during the S/G2 phase of cell cycle when the sister chromatid is available, while c-NHEJ works independently of sequence homology dominantly in G1 [13, 14]. The choice of DSB repair pathways is mainly governed by the opposing activities of two key regulators TP53-binding protein 1 (53BP1) and breast cancer susceptibility protein-1 (BRCA1) that promote c-NHEJ and HDR, respectively [12, 15]. Emerging evidence indicates that SETD8 and relevant methylation states of histone H4K20 oscillate during the cell cycle with major implications for choice of DSB repair pathways, especially for the recruitment of 53BP1 and BRCA1, linking the DNA damage repair mechanisms to SETD8 activity and its corresponding H4K20 methylation [16, 17].

In this review, we discuss the progress made to date in elucidating the mechanisms by which the lysine mono-methyltransferase SETD8 functions in DNA damage repair and its therapeutic relevance. We will lay a major emphasis on the role of SETD8 and its corresponding disparate H4K20 methylation states in the recruitment of 53BP1/BRCA1, two key determinants in the choice of DSB repair pathways, to DNA damage sites and highlight areas that remain to be defined.

## The activity and substrates of SETD8

### The characterization of SETD8 as a sole mono-methyltransferase in mammalian

**Fig. 1:**
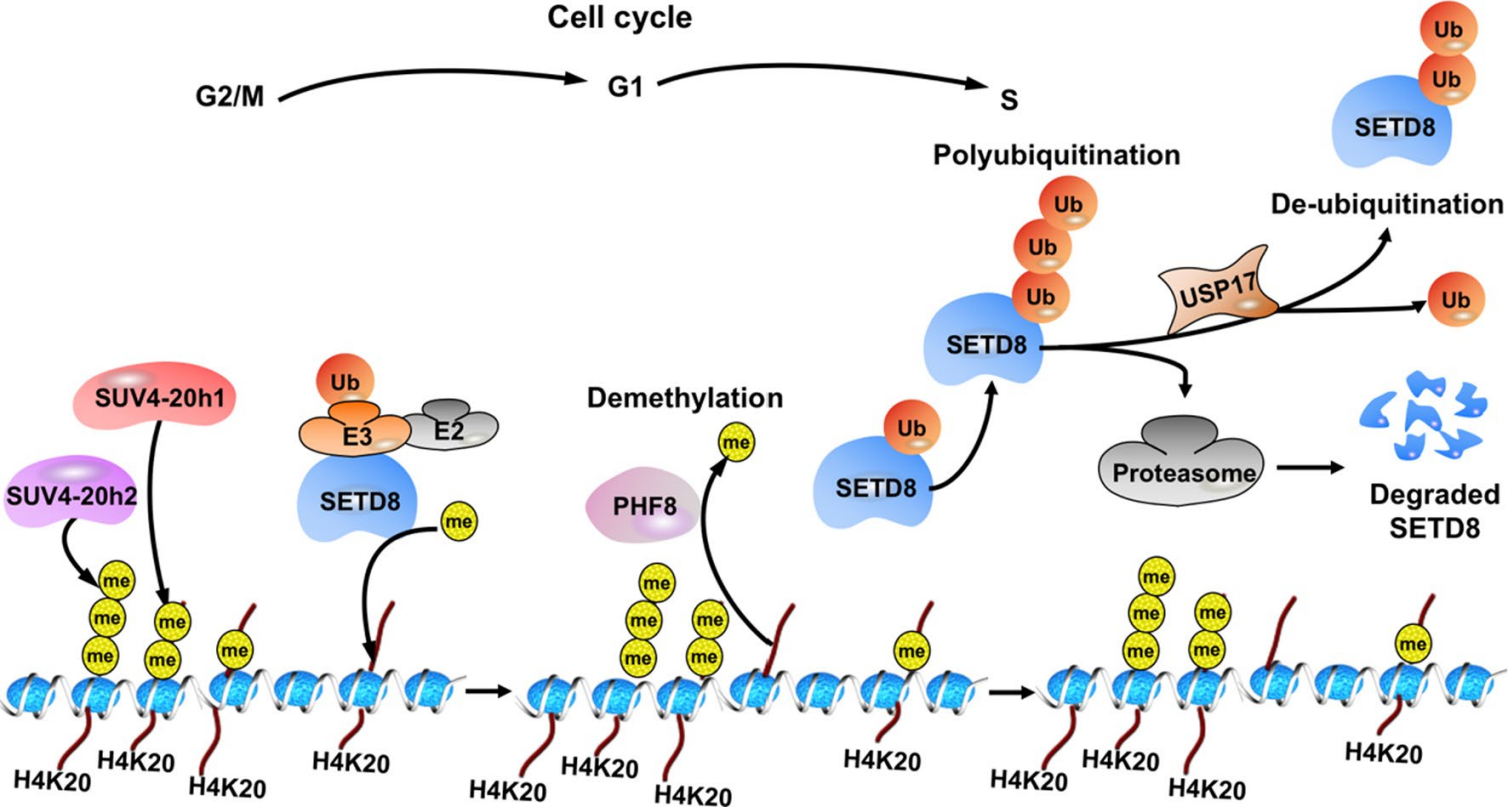
The methylation activity and regulatory network of SETD8. SETD8 monomethylates histone H4 to generate H4K20me1 on chromatin, a prerequisite for di- and tri-methylation catalyzed by SUV4-20h1 and SUV4-20h2 to produce H4K20me2 and H4K20me3, respectively. Conversely, PHF8 regulates the demethylation of H4K20me1. The levels of SETD8 are downregulated by the ubiquitin–proteasome system. The deubiquitinating enzyme USP17 is reported to stabilize SETD8 by removing polyubiquitin chains from (Ub)_n_-SETD8

SETD8 (also known as PR-SET7, SET8, and KMT5A), located on chromosome 12q24.31, belongs to a family of methyltransferases which contain the characteristic Su(var)3–9, Enhancer of Zeste, Trithorax (SET) domain [8, 18, 19]. To date, more than 50 SET domain containing proteins or potential histone lysine methyltransferases (HKMTs) have been identified in the eukaryotes [3, 19, 20]. Apart from Dot1/ DOT1L, all HKMTs possess a SET domain with 130 amino acids [4, 21]. SETD8, originally identified in 2002, has long been known as a methyltransferase that catalyzes H4K20me1 exclusively to maintain silent chromatin, and upon which the other enzymes, such as SUV4-20h1 and SUV4-20h2, catalyze further methylation to generate H4K20me2 and H4K20me3 (Fig. 1) [8, 19, 22]. The methyltransferase multiple myeloma SET domain (MMSET)/Wolf-Hirschhorn syndrome candidate 1 (WHSC1) is recently reported to be another di-methyltransferase that catalyzes H4K20me2 in DSBs repair [23, 24]. Conversely, removal of the methyl moiety is carried out by a group of ‘erasers’ [7, 25–28]. The PHD Finger Protein 8 (PHF8) is a demethylase, that contains PHD and Jumonji C domains, with a wide spectrum of substrates, including H4K20me1, H3K9me1/2 and H3K27me2 [25]. Unlike this, the other members of this family PHD Finger Protein 2 (PHF2) and DPY-21 prefer to demethylate H4K20me3 and H4K20me2, respectively [25, 27]. hHR23A has been also identified as a histone H4K20 demethylase [28]. Moreover, H4K20 exerts different effects dependent on disparate methylation states [5, 16, 17, 29–31]. H4K20me1 associates with chromatin condensation which impacts DNA replication, cell cycle progression and the DNA Damage Response (DDR) [8, 31, 32]. H4K20me2 is a prevailing methylation state in interphase related to guidance of DNA repair proteins mainly to euchromatin flanking DNA damage, whereas H4K20me3 is a marker that correlates with transcriptional repression in heterochromatic domains [3, 17, 33].

Recently non-histone proteins, such as p53, proliferating cell nuclear antigen (PCNA) and Numb, are reported to be among substrates of SETD8 [20, 34, 35]. Although other yet-to-be-identified substrates may exist, SETD8 specifically localizes to mitotic chromosomes and prefers nucleosomal substrates [22–24]. Importantly, loss of SETD8 causes embryonic lethality suggesting its vital role in tissue development [18]. Also, an increasing number of studies in recent years suggest the requirement of SETD8 for senescence and tumorigenesis, placing SETD8 and its relevant H4K20 methylation at central nodes of many important pathways [4, 36, 37].

### SETD8 and its predominant substrate H4K20

First isolated by Nishioka et al. [8], SETD8 is found as a mono-methyltransferase in mammalian that catalyzes the methylation of histone H4K20 to maintain silent chromatin by precluding the adjacent acetylation of histone H4 on lysine 16 (H4K16Ac), which is a prevalent and reversible posttranslational modification that directly contributes to decondensed state of chromatin [8, 38]. Meanwhile, the same researcher described distinct patterns of SETD8 activity and H4K20 methylation during cell cycle progression [39]. Several studies later substantiate both protein levels of SETD8 and H4K20me1 fluctuating during the cell cycle due to ubiquitin-dependent destruction of SETD8 [3, 18, 40]. In the G2/M phase, SETD8 associates with the mitotic chromosomes to give rise to H4K20me1 marks on chromatin, while as cells progress through G1 phase, SETD8 proteins reduce progressively and then disappear at the onset of DNA replication [3, 18]. Apart from cell cycle progression, SETD8 and its corresponding H4K20 methylation have a well-documented regulatory role in gene transcription, DNA replication, and the DDR [5, 16, 17, 29, 30].

Further studies also uncovered the preference of nucleosomes over free histones as substrates for SETD8 methylation, thereby raising the questions: how the enzyme interacts with nucleosomes and chromatin factors [20, 34, 35, 41]. Extensive studies subsequently have attempted to understand the mechanisms underlying its substrate specificity from different perspectives [32, 42–44]. Early structural analyses attributed the substrate specificities of SETD8 to the Phe/Tyr switch, which alters the affinity of an active-site water molecule, and the Phe/Tyr switch mutant altered the substrate specificity from a mono-methyltransferase to a di-methyltransferase [42]. Another study reported the mechanism of SETD8-mediated H4K20 mono-methylation [43]. Using the kinetic isotope labeling, they showed an early transition state of SETD8, i.e., the S-adenosyl-L-methionine-SETD8-H4K20 intermediate complex. These observations are important for the development of selective transition-state inhibitors that could block SETD8 [43]. Girish et al. [44] also provide structural insights into the specificity and demonstrate that a basic N-terminal extension of SETD8 determinates nucleosome binding. It interacts with nucleosomal H2A/H2B histone dimer “acidic patch” and likely the nucleosomal DNA to anchor SETD8 to the nucleosome substrates [44]. It is a conformational change that positions the catalytic site of SETD8 neighboring the targeted methylation site for the subsequent H4K20 methylation [44]. More recently, the nucleophilic amino group of lysine is verified to be central for histone lysine methyltransferase catalysis [32].

Collectively, the findings underscore H4K20 as a major effector of SETD8 and a crucial mediator of its impact on cellular biology. Despite still a long way to go before how SETD8 and H4K20 exactly interact with each other being entirely clear, these findings are of significance in drug development targeting SETD8, which is closely related to tissue development, senescence and tumorigenesis [4, 42, 45].

### SETD8 and other targets

Besides H4K20, SETD8 also has been found to be able to modify non-histone proteins, including PCNA [20], p53 [34] and Numb [35]. Evidence suggests that the tumor suppressor protein p53 is a substrate of SETD8, which specially monomethylates p53 at lysine residue 382 [34]. PCNA is an evolutionarily well-conserved protein which ubiquitously expresses in all species [20]. SETD8 regulates the stability of PCNA protein by methylation at lysine 248, which enhances the interaction between PCNA and the flap endonuclease FEN and thereby promotes cell proliferation [20, 46]. Numb exerts a proapoptotic activity through its interaction with p53 and is identified as a novel substrate of SETD8 [35]. It is methylated in its phosphotyrosine-binding (PTB) domain by SETD8 to uncouple Numb from p53, resulting in an increase in p53 ubiquitination and degradation [35]. It is possible that along with the deep-going research, more as-yet-unidentified substrates may emerge in the near future and it will provide further insights into the function of SETD8 and its role in the disease development, in particular tumorigenesis.

## Roles for the methyltransferase SETD8 in DNA damage repair

### 53BP1/BRCA1 dictates choice of DSBs repair pathways

Cytotoxic DSBs may result in deleterious consequences including genomic instability and cancer origin, when failure to be detected and repaired faithfully [9, 11, 12]. There are four DNA damage repair pathways employed for DSBs repair, including HDR, c-NHEJ, single-strand annealing (SSA), and an alternative error-prone DSB repair pathway named alternative end-joining (alt-EJ) [14, 23]. Among these, the error-free HDR and error-prone c-NHEJ are two most predominant DSBs repair mechanisms, the choice of which is influenced by multi-layered factors [9, 10, 12, 13]. One determinant is the nature of the DSB end since accurate HDR requires a 3′ overhang to initiate the critical strand invasion, while c-NHEJ only promotes the potentially inaccurate ligation of blunt DNA ends or DSBs with short overhangs [9, 10, 12, 45]. As a consequence, c-NHEJ is inhibited by end resection which generates single-stranded DNA (ssDNA) based on 5′-3′ nucleolytic degradation [9, 10, 45]. On the other hand, the phase of cell cycle when DSBs occurs is another major determinant for which pathway to be triggered [9]. If the DSBs form in S/G2 phase, it is usually repaired by the accurate HDR. In contrast, c-NHEJ becomes a preferred DSB repair pathway in G1 phase [9]. Such a decision must integrate information about the nature of DSB end, phase of cell cycle and other yet-to-be-determined factors to prevent unnecessary and potentially deleterious alterations [45].

Recently, 53BP1 and BRCA1 have been elevated to be master determinants for DSBs repair pathway choice, since they function at the intersection of two major pathways with opposing activities [9, 10, 15]. Most studies have shown that 53BP1 stimulates c-NHEJ, whereas BRCA1 promotes the end resection and HDR [12, 15]. The CtBP-interacting protein (CtIP) and its downstream effector and anti-resection regulator Rap1-interacting factor 1 (RIF1) are identified as important factors to regulate 53BP1/BRCA1-mediated choice of the repair pathway [15, 45, 47]. Recent evidence further suggests that the choice of repair pathway is controlled by a cell cycle-regulated inhibitory circuit composed of 53BP1-RIF1 and BRCA1-CtIP [15, 45]. 53BP1, together with RIF1, favors c-NHEJ in G1 phase by opposing BRCA1-dependent HDR, while BRCA1-CtIP prefers HDR in S/G2 phase through antagonizing 53BP1/RIF1 [9]. The outcome of this battle for DSBs repair pathway choice at the break site is ultimately dominated by cell cycle position [45]. However, 53BP1 is also required in G2 phase for HDR when the DSBs occur in heterochromatin, in which case 53BP1 promotes the formation of phosphorylated KRAB-associated protein 1 (KAP-1) foci [48]. These findings collectively emphasize 53BP1 and BRCA1 as the dominant factors controlling the choice of DSBs repair pathways.

### Potential link between SETD8 and 53BP1/BRCA1-mediated DSB repair pathways

The DDR machinery orchestrates an intricate network of DNA damage and repair signaling and proteins to detect, recruit and repair DNA lesions, including DSBs [9–11, 49]. A broad spectrum of posttranslational modifications has been implicated in the DDR [11, 12, 47, 50]. Phosphorylation is found one of the earliest events in DSBs repair [10, 11]. The ataxia-telangiectasia mutated kinase (ATM), ataxia-telangiectasia Rad3‐related kinase (ATR), and/or the DNA dependent protein kinase (DNA-PK) belonging to the phosphatidylinositol 3-kinase-like protein kinase (PIKKs) family are reported to be involved in the DDR [10]. The histone variant H2AX can be phosphorylated by kinases ATM, ATR and/or the DNA-PK at serine 139 to form γH2AX, an important DDR marker that serves as a scaffold for accumulation of large signaling and repair protein complexes [9, 11]. Moreover, a set of ubiquitylations by the E3 ubiquitin ligases, such as the ring finger protein 8 (RNF8) and the ring finger protein 168 (RNF168), take place as well to promote the DDR signaling cascade through mediating 53BP1, an important factor in DSB repair [11, 12, 47, 50]. In addition, methylation has also been reported in the DDR [11, 51].

There is an increasing body of evidence to link the methyltransferase SETD8 to the DSBs repair [2, 11, 18]. In 2007, two papers have pointed out the importance of methyltransferase activity of SETD8 in S phase progression and the DSBs/DDR [2, 18]. Jorgensen et al. [2] documented that inhibition of SETD8 suppresses cell proliferation following induction in DSBs. Meanwhile, Tardat et al. [18] also showed massive DSBs with subsequent robust DDR trigged by the SETD8-depletion related stress. SETD8 is also proposed to be transiently recruited to DSBs loci preceding that of 53BP1, and depletion of SETD8 reduces 53BP1 foci, suggesting that the recruitment of 53BP1 depends on SETD8 [11, 46]. The following experimental researches substantiate that SETD8 promotes DSBs repair via the c-NHEJ pathway and the SETD8 mutant in its catalytic domain does not recruit 53BP1 when DSBs occur [11, 46]. This collectively underscores the importance of the methyltransferase activity of SETD8 in 53BP1-mediated c-NHEJ repair pathway. Recently, studies further indicate that disparate states of H4K20 methylation mediates recruitment of 53BP1 and BRCA1, which dominates the choice of DSB repair pathways and further links SETD8 to the DSBs repair [16, 17]. Besides, SETD8 is also reported to be involved in checkpoint regulation through regulating the methylation of its non-histone substrates, such as p53, PCNA and Numb [6, 34, 35, 52] (Table 1).

**Table 1:** Roles for the methyltransferase SETD8 in DNA damage repair

Roles of SETD8	Mechanism	References
DNA damage	Inhibition of SETD8 expression induces massive DSBs	[2, 11, 18, 40]
	Inactivation of the CRL4-Cdt2-PCNA-SETD8 degradation axis leads to DNA damage	[52]
	SETD8 catalyzes PCNA methylation on lysine 248 that enhances its interaction with FEN1, whereas loss of PCNA methylation induces DNA damage and makes cells more susceptible to DNA damage	[20]
	The E3 ubiquitin ligases RNF168 mediates SETD8 localization to chromatin flanking DNA damage	[53]
	Removal of SET8 supports the modulation of chromatin structure after DNA damage	[54]
53BP1 recruitment	H4K20me2 is a docking site for 53BP1	[33]
	The SUV4-20 activity and H4K20me2/3 methylation are inessential for recruitment of 53BP1 and c-NHEJ-directed repair pathway	[55]
	SETD8-mediated H4K20me1 alone is insufficient, but H4K20me2 is also required, for 53BP1 binding and the DSBs repair	[56–58]
	53BP1 recruitment depends on H4K20me2 established prior to DNA damage rather than de novo H4K20 methylation mediated by MMSET/WHSC1, and acetylation at H4K16 inhibits 53BP1 binding to extant H4K20me2	[59]
	Replication-coupled dilution of H4K20me2 guides 53BP1 to pre-replicative chromatin	[17]
	SETD8 interacts with RNF8 and RNF168 in a ubiquitination-dependent manner that promotes H2A ubiquitination in response to DNA damage and 53BP1 is a reader of the DNA damage-induced H2A Lys 15 ubiquitin mark	[60, 61]
	SETD8 is transiently recruited to laser-induced DNA damage sites through its interaction with PCNA, which promotes 53BP1 recruitment to the DSBs	[46]
	The histone methyltransferase MMSET/WHSC1 catalyzes H4K20me2 based on SETD8-mediated H4K20me1, which facilitates 53BP1 recruitment in response to DSBs	[23]
	SETD8 is functionally required for 53BP1 accumulation and for efficient repair of DSBs specifically via the NHEJ	[11]
	The SETD8 inhibitor UNC-0379 blocks H4K20 methylation and reduced recruitment of the 53BP1 protein to DSBs	[55]
	The methyltransferase MMSET-mediated H4K20me2 recruits the nucleotide excision repair factor XPA to DNA loci in a 53BP1-dependent manner	[24]
BRCA1 recruitment	H4K20me0 recognition is required for TONSL–MMS22L binding to chromatin and accumulation at challenged replication forks and DNA lesions	[62]
	BRCA1 recruitment requires recognition of H4K20me0, linking DSB repair pathway choice directly to sister chromatid availability	[16]
	BRCA1-BARD1 binds nucleosomes through recognition of both unmethylated H4K20 and H2AK15ub to promote HR-mediated DSB repair	[63]
	Recognition of monoubiquitin at the N terminus of H2A by BRCA1-BARD1 promotes ubiquitylation at the C terminus of H2A, which recruits SMARCAD1 to oppose the positioning of 53BP1	[64]
	RNF168-mediated localization of BARD1 recruits the BRCA1-PALB2 complex to DNA damage	[65]
Checkpoint regulation	SETD8 catalyzes p53 methylation and deletion of SETD8 arguments the checkpoint activation functions of p53	[34]
	Inactivation of the CRL4-Cdt2-PCNA-SETD8 degradation axis increases expression of p53 and its transactivated proapoptotic genes	[52]
	SETD8 mediates Numb methylation that uncouples Numb from p53, increasing p53 ubiquitination and degradation	[35]
	SETD8 abundance regulated by SCF^b−TRCP^-mediated pathways contribute to the onset of DNA damage-induced checkpoints	[6]

### Roles of H4K20 di-methylation in 53BP1-mediated DSB repair pathways

#### The 53BP1 domains and its recruitment upon the DSBs

53BP1 is a critical component in the DDR as well as a necessary and pivotal determinant of c-NHEJ-directed DSB repair [10, 12]. It rapidly forms large foci near DNA lesions where ATM- or ATR-mediated DNA damage signaling is activated [50]. While DNA end resection is a prerequisite for HDR, 53BP1 shields DNA lesions against excessive 5′ end nucleolytic digestion to promote c-NHEJ [12]. Restrained resection is also achieved by 53BP1‐dependent recruitment of RIF1, REV7 and the Shieldin complex, which may also promote recruitment of Ku70/80, the important c-NHEJ factor [12, 49]. The factors that dictate the recruitment of 53BP1 appear to be complex [12, 15, 49, 51]. SETD8 has been reported as a required factor for accumulation of 53BP1 at chromatin flanking DSBs [11, 46]. Since SETD8 is a cell cycle-regulated protein that is nearly absent in S phase, it may be puzzling how SETD8 mediates the accumulation of 53BP1 across all stages of the cell cycle [30, 40]. In fact, compelling evidence indicates that recruitment of 53BP1 depends on the catalytic activity of SETD8, by which H4K20 is methylated in an evolutionarily conserved manner among diverse organisms [17, 46, 52].

**Fig. 2:**
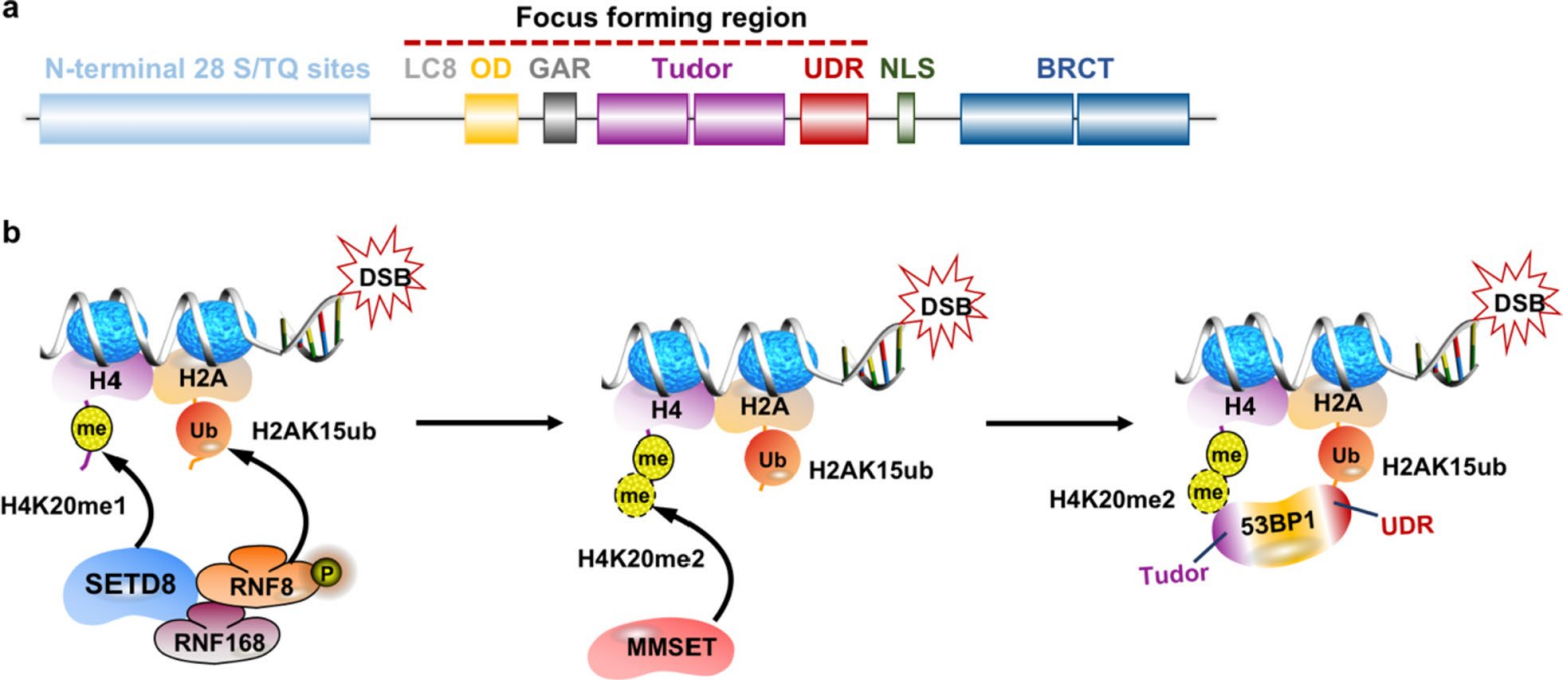
SETD8 contributes to 53BP1 recruitment in DSBs repair. (**a**) Domain structure of human 53BP1. The central focus forming region (FFR) is the minimal region required for the accumulation of 53BP1, comprising the oligomerization domain (OD), a glycine–arginine-rich (GAR) motif, the tandem Tudor domain, a ubiquitin-dependent recognition (UDR) motif, and the dynein light chain (LC8) binding domain, and (**b**) SETD8 contributes to 53BP1 recruitment in DSBs repair. The UDR motif mediates the recruitment of 53BP1 to the nucleosomes containing H2AK15ub, while SETD8 catalyzes the methylation of histone H4K20 upon the DSBs which is bound by the tandem Tudor domain of 53BP1. In addition to catalyzing H4K20 methylation, SETD8 also interacts with the E3 ubiquitin ligases RNF8 and RNF168 to promote H2AK15ub formation in response to DSBs. Another assumption is that H4K20me2 can be increased locally by the methyltransferase MMSET which localizes at the DNA damage foci since no obvious accumulation of SUV4-20h1/2 is observed upon the DSBs

The importance of H4K20 methylation in DNA damage repair was originally demonstrated in the fission yeast [66]. The human 53BP1 is a homolog of yeast crumbs cell polarity complex component 2 (Crb2) protein [66]. In the mammals, the tandem Tudor domain, which recognizes methylated lysine residues, is conserved in 53BP1 [9]. It recognizes histone H4K20 methylation and allows the binding of 53BP1 (Crb2) to the DSBs [9, 33]. The central focus forming region (FFR) of 53BP1, where the Tudor domain located in, is the minimal region required for its accumulation at DSBs-flanking chromatins [9]. Apart from tandem Tudor domain, the FFR also has several other functional domains, such as the oligomerization domain (OD), a glycine/arginine-rich (GAR) motif and a ubiquitin-dependent recruitment (UDR) motif (Fig. 2a) [9].

The UDR motif and tandem Tudor domain, proximal to each other in position, are involved in 53BP1 accumulation [9, 11]. On the one hand, the UDR motif mediates 53BP1’s recruitment to the nucleosomes containing H2A ubiquitination at Lysine 15 (H2AK15ub) generated from a sequential ubiquitylation by the E3 ubiquitin ligases RNF8 and RNF168 [61, 67]. Recently, SETD8 has been reported to interact with RNF8 and RNF168 to promote H2A ubiquitination in response to DNA damage, while RNF8 and RNF168 mediate the localization of SETD8 to the DSBs in a ubiquitination-dependent manner [53, 60]. As mentioned already, a structural analysis revealed that the interaction between H2A/H2B dimer “acidic patch” and SETD8 ensures the DSB-dependent recruitment of SETD8 to the nucleosome [44]. It is likely that the RNF8-mediated SETD8 ubiquitination can also facilitate the localization of RNF168 to the H2A for its subsequent ubiquitination [44, 60]. The phosphorylation of histone variant H2AX occurs only on chromatin close to DNA lesions where ATM/ATR signaling is activated [50, 61]. When MDC1 docks via its BRCT domains to γH2AX, its subsequent phosphorylation serves to recruit the E3 ubiquitin ligase RNF8, which mediates ubiquitylation of linker histone H1, SETD8, and the following recruitment of RNF168 that further ubiquitylates to form H2AK15ub [60, 67–69]. 53BP1, as a histone modification reader, recognizes H2AK15ub induced by DNA damage [61]. On the other hand, the tandem Tudor domain recognizes chromatins with histone H4 specially di-methylated at Lys20 (H4K20me2) [33]. The E3 ubiquitin ligase RNF8 is proposed to be able to destroy the competing proteins of H4K20me2, such as the malignant-brain-tumor (MBT) protein L3MBTL1 (L3MBTL1) and JMJD2A at DSBs, to make H4K20me2 accessible to 53BP1 [11, 30, 33, 70]. Collectively, it is presumed that the stable accumulation of 53BP1 at chromatin flanking DSBs is dependent on recognition of H4K20me2 by its Tudor domain, which requires the simultaneous engagement of H2AK15ub by the UDR motif (Fig. 2b) [9, 61]. However, there are also studies indicating that 53BP1 could form transient foci in cells absent of H2AX [50, 61]. The c-NHEJ deficiencies caused by loss of 53BP1 are more pronounced than those found lacking H2AX [61]. Therefore, it is likely that the H2AX-independent mechanisms underlying 53BP1 accumulation at DSBs operate in a different context.

#### Role of H4K20me2 in 53BP1 recruitment and c-NHEJ-directed repair

Histone H4K20 methylation, catalyzed by several enzymes to generate different states of methylation, is known to oscillate during the cell cycle with diverse epigenetic functions [2, 16, 17, 29, 31, 40]. As well, H4K20me1 is exclusively methylated by SETD8 and is considered as a prerequisite for generation of di- and tri-methylation of H4K20 (H4K20me2/3) [56, 71]. Prior studies have established that SETD8 deficiency in catalytic domain induces the DSBs and corresponding 53BP1 recruitment, implicating the crucial role of SETD8 activity and H4K20 methylation in 53BP1-mediated DSBs repair [2, 11, 18]. However, it is still uncertain which methylation state of H4K20 dictates the recruitment of 53BP1 to DSBs among the three disparate states of H4K20me1/2/3. A study has found that primary mouse embryonic fibroblasts (pMEFs) depleted of *Suv4-20h* induce significant decrease in levels of H4K20me2/3, but only minor defects in DSBs-elicited 53BP1 localization, suggesting inessential of SUV4-20 activity and H4K20me2/3 methylation for recruitment of 53BP1 and c-NHEJ-directed repair pathway [71]. But other studies argue against this model [56–58]. Depletion of SUV4-20h1/h2 in HeLa cells is substantiated to cause an increase in H4K20me1 with a concurrent reduction in levels of H4K20me2 remarkably, which impairs 53BP1 binding to DSBs loci, indicative of that H4K20me1 alone is insufficient, but H4K20me2 is also required, for 53BP1 binding and the DSBs repair [56]. Accordingly, 53BP1 localization prevented by blocking H4K20me2/3 through the SUV4-20 inhibitor A-196 provides evidence as well for the importance of SUV4-20 activity in 53BP1 recruitment and efficient DSB repair [58]. These cumulative data echo the findings that either SETD8 or SUV4-20h1 deficient cells show a reduction in DSBs-induced accumulation of 53BP1, emphasizing the orchestrated and concerted activities of both SETD8 and SUV4-20h1 as dominants of 53BP1 relocation to DNA damage sites and proper c-NHEJ-directed repair [57].

H4K20me2 is the major methylation state of H4K20, accounting for over 80% of total histone H4, in eukaryotic cells [71]. Although it is clearly a docking site for 53BP1, the recognition that H4K20me2, as a prevalent mark across the genome fails to increase markedly in overall levels upon DNA damage, does not explain why 53BP1 is only recruited to DSBs-flanking chromatins [17, 33, 61]. Early evidence suggests that 53BP1-recruitment is achieved through exposure of pre-existing H4K20me2 to a large extent via chromatin structure remodeling [40, 71]. It is showed that H4K20me2 is highly abundant and prevalent on chromatins largely in the absence of DNA damage, leaving only a small pool of available substrate to modify after damage [71]. Besides, a possible model has been issued in fission yeast that DSBs induce exposure of existing H4K20me2 previously hidden or buried in the context of packed chromatin for Crb2 binding [72]. Various chromatin remodeling factors, including KAP-1 and RNF168, have been implicated in this dynamic chromatin relaxation process, yet very little experimental evidence has been provided [48, 69, 73]. Recent data also show that in eukaryotes, 53BP1 binding to extant H4K20me2 is antagonized by H4K16 acetylation until its transient and localized deacetylation induced by the DSBs, suggesting 53BP1 foci assemble primarily on H4K20me2 generated prior to DNA damage rather than de novo H4K20 methylation elicited by the DSBs [59].

On the other hand, supportive evidence is also in position for the assumption of de novo H4K20 methylation [23, 46, 74]. Either depletion of SETD8 or inactivation of its methyltransferase activity is found disrupting the recruitment of 53BP1 to DNA damage sites, which links de novo H4K20 methylation to 53BP1 localization [46]. Moreover, it is also proposed that H4K20me2 can be increased locally by the methyltransferase MMSET/WHSC1 that localizes at the DNA damage foci since no obvious accumulation of SUV4-20h1/2 is observed upon the DSBs [23]. Recent experiments have substantiated that MMSET generates H4K20me2 and H3K36me2 marks depending on the chromatin context [24]. DICER mediates the recruitment of the MMSET to the DNA damage site, which catalyzes H4K20 di-methylation that contributes to subsequent recruitment of the nucleotide excision repair factor XPA to DNA loci in a 53BP1-dependent manner [74]. It can also be considered as supportive evidence for the hypothesis of de novo methylation. Therefore, more studies are still needed to uncover the mechanism underlying H4K20 methylation-mediated 53BP1 localization, not only helpful to understand the mechanism of disorders related to H4K20-53BP1 axis, but also to develop the effective therapeutic strategies that targeting DNA damage repair pathways.

### Roles of unmethylated H4K20 in BRCA1-mediated DSB repair pathways

#### The characterization of BRCA1 and its functional domains

The tumor suppressor BRCA1 is initially discovered as an early-onset breast cancer susceptibility gene and its mutation predisposes individuals to early onset of disparate familial diseases, leading to not only breast and ovarian cancers, but also the Fanconi anemia (FA) [75, 76]. Importantly, a substantial fraction of breast tumors arising in women carrying BRCA1 mutations are triple negative, representing one of the most aggressive forms of the disease [77]. Compelling evidence has shown that the antitumorigenic properties of BRCA1 mainly stem from its role in repair of DSBs and maintenance of replication forks [78, 79]. Corresponding to 53BP1, BRCA1 is considered another critical regulator in the DDR to promote HDR-mediated DSBs repair [78, 80, 81]. BRCA1 and its obligate partner BRCA1-associated RING domain protein 1 (BARD1) heterodimerize to form BRCA1-BARD1 to facilitate the initial nucleolytic resection of DNA lesions which generates a single-stranded template and participates in the recruitment and regulation of another tumor suppressor complex breast cancer susceptibility gene 2 (BRCA2)-PALB2 and the recombinase RAD51 [52, 78]. More importantly, emerging evidence indicates that the unmethylated state of histone H4K20 (H4K20me0) is required for BRCA1 recruitment to DNA damage sites to tune the HDR-directed repair pathway [16].

**Fig. 3:**
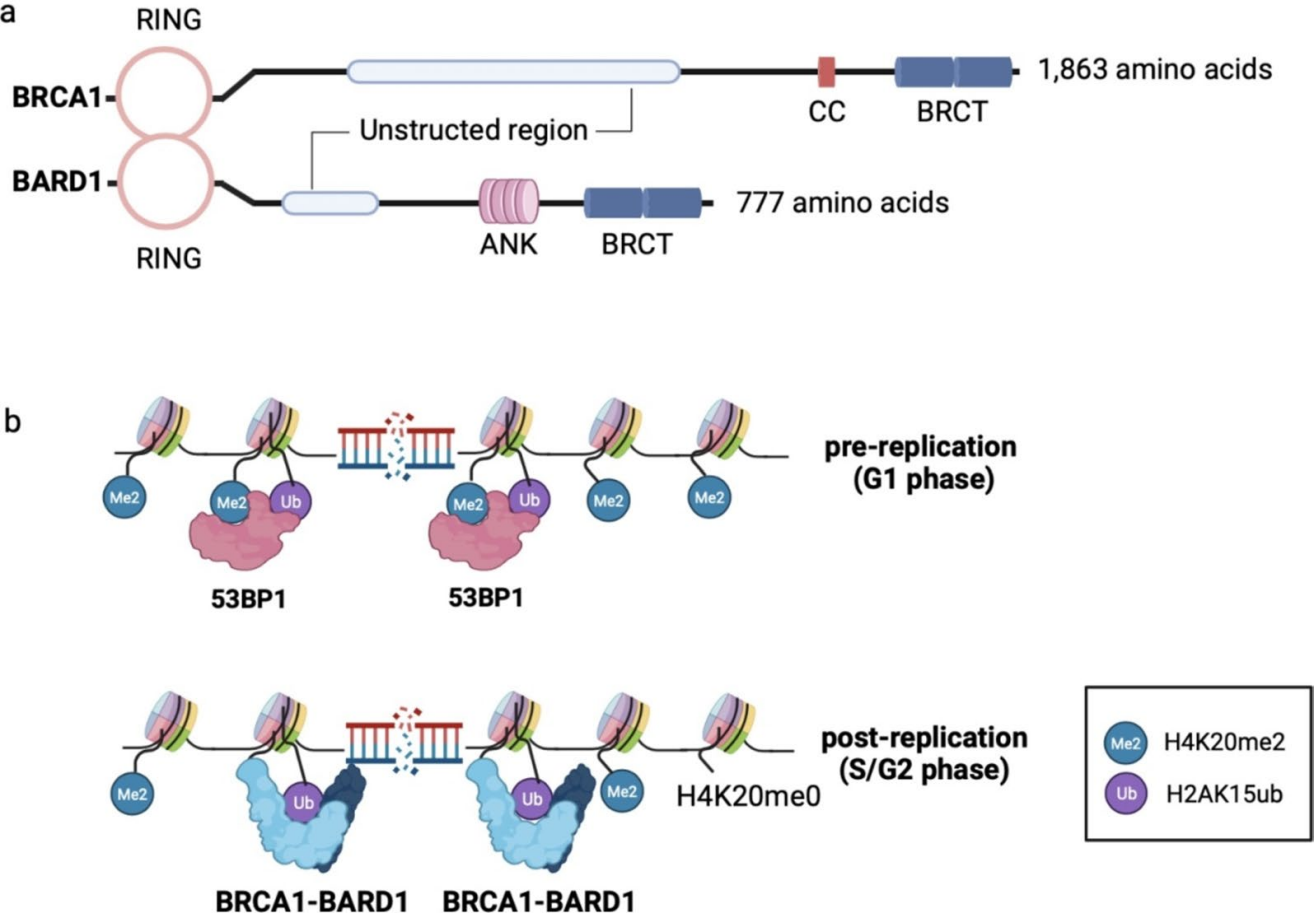
The functional domains of BRCA1-BARD1 and proposed model for bivalent nucleosome recognition by BRCA1/53BP1. (a) The functional domains of BRCA1 and BARD1. The RING domains of BRCA1 and BARD1 mediate their heterodimerization and is crucial for the E3 ubiquitin ligase activity of the BRCA1-BARD1 complex (b) Proposed model for 53BP1 and BRCA1 recruitment by bivalent nucleosome recognition. Their recognitions of shared H2AK15ub-modified nucleosomes, but distinct methylation states of H4K20, ensure their respective preference for chromatins in a DSB-dependent-specify and a cell cycle-dependent-specify, respectively, which dictates choice of DSB repair pathways

BRCA1 is a large protein of 1863 amino acids with several functional domains, including two BRCA1 C-terminal (BRCT) repeats, a coiled-coil (CC) domain, a really interesting new gene (RING) domain and an unstructured region (Fig. 3a) [76, 80, 82]. BRCA1 exists as a heterodimer with the BARD1 tumor suppressor, a protein of 777 amino acids, which possesses a RING domain, four ankyrin (ANK) repeats, two BRCT domains, as well as an unstructured region (Fig. 3a) [83]. BRCA1 and BARD1 heterodimerize with E3 ubiquitin ligase activity through their respective RING domain, which has been lately proved to be promoted by a sirtuin deacetylase SIRT2 [84]. The BRCT is a phospho-protein binding domain that mediates interactions with different partner proteins involving in the DDR [76, 79, 81, 85, 86]. The BRCT domain of BRCA1 interacts with CtIP in a cell cycle-dependent manner, which is involved in DNA end resection to generate ssDNA [86]. Whereas the BRCT domain at the C-terminal of BARD1 mediates its association with poly (ADP-ribose) (PAR) that is synthesized by poly (ADP-ribose) polymerase, and with heterochromatin protein 1 (HP1), a prerequisite for formation and maintenance of heterochromatin [76, 79, 81, 85]. The unstructured regions of BRCA1 and BARD1 are both indispensable for binding DNA and interacting with RAD51 to enhance its recombinase activity [78]. Substantial evidence emphasizes that the RING dimer and the BRCT domains are crucial for the tumor suppression activity of BRCA1-BARD1 [87, 88]. Besides, the CC domain and ANK repeats are unique features of BRCA1 and BARD1, respectively [28, 80, 89]. The CC domain of BRCA1 mediates the direct binding of BRCA1 and PALB2, a partner and localizer of BRCA2, which serves as a molecular scaffold for formation of the BRCA1-PALB2-BRCA2 complex [80]. On the other hand, the ANK repeats of BARD1 facilitate localization of BRCA1-BARD1 complex to DSBs loci through specific recognition of the unmethylated histone H4K20, which marks nucleosomes of newly replicated DNA [16]. This implies that H4K20 methylation states potentially mediate recruitment of BRCA1 in a cell cycle-specify manner.

#### Role of unmethylated H4K20 in recruitment of BRCA1 and HDR-directed repair

The opposing activities of BRCA1 and 53BP1 dictate pathway choice of DSBs repair [10, 11, 16]. Evidence suggests that BRCA1-BARD1 promotes HDR-directed DSBs repair by antagonizing 53BP1, which is recruited through bivalent binding to H2AK15ub and H4K20me2 in a DSB-dependent manner to facilitate c-NHEJ [16, 17, 61]. But how BRCA1 complex antagonizes 53BP1 to promote DNA resection and HDR-directed repair is a long-standing problem. An explanation is that BRCA1-BARD1 monitors the replicative state of the genome to oppose 53BP1 function, routing only DSBs within sister chromatids to HDR [16]. H4K20me0 is specific on histones newly incorporated during DNA replication and marks the post-replicative genomic locus until G2/M, when a surge of SETD8 methyltransferase activity catalyzes H4K20me1 [62]. BRCA1-BARD1 complex thereby identifies post-replicative chromatin by recognition of H4K20me0 through the reader ANK repeats of BARD1, which prevents 53BP1 access to H4K20me2 at DSBs loci and promotes resection only when a sister chromatid is available for HDR [16]. It is supported by the finding that forced methylation of H4K20 by non-degradable SETD8 blocks BRCA1 recruitment and RAD51 loading at DSBs in S phase [17]. On the other hand, there is also another model proposed to explain how BRCA1 complex is recruited to chromatin in a DSB-dependent manner, which underscores the necessity of the ubiquitin ligase activity of RNF168 [65]. It is suggested that BRCT domain ubiquitin-dependent recruitment motif (BUDR) of BARD1 recognizes H2A-associated ubiquitin modifications catalyzed by RNF168, followed by recruitment of PALB2-RAD51 to DSBs sites via the CC domain-mediated BRCA1-PALB2 interaction [65]. These findings echo a study that BARD1 mediates localization of BRCA1 complex to nucleosomes proximal to DSBs through bivalent interactions [85]. The BUDR and ANK repeat domain of BARD1 recognize H2AK15ub and H4K20me0, respectively, to ensure high-affinity binding of BRCA1-BARD1 complex to post-replicative chromatin flanking DSBs, which facilitates HDR and antagonizes 53BP1-mediated pathway, establishing a simple paradigm for the control of the DSB repair pathway choice (Fig. 3b) [63]. A recent study has revealed that BRCA1-BARD1 recognizes monoubiquitin at the N terminus of H2A that blocks the formation of polyubiquitin chains and promotes ubiquitylation at the C terminus of H2A [64]. The ubiquitylation at H2A tail recruits the chromatin remodeler SMARCAD1, which opposes the positioning of 53BP1 [90]. This may provide a possible explanation for the antagonistic relationship between BRCA1 and 53BP1 recruitment. Besides, defects in either H4K20me0 or H2AK15ub recognition sensitize cells to PARP inhibition, which provides a circumstantial evidence for contribution of epigenetic modifications on both histone H2A and H4 tails to chromatin engagement of BRCA1 complex, and implies a therapeutic strategy targeting BRCA1-mediated HDR repair, especially targeting the recruitment process of BRCA1 [16, 63, 65].

Collectively, evidence indicates that recruitment of 53BP1 and BRCA1 complexes that converge at DSBs sites requires binding to both histone H2A and H4 tails, through recognition of shared mono-ubiquitination of histone H2AK15 and distinct methylation states of H4K20 [17, 61, 63]. The cross-talk between HDR and c-NHEJ pathways remains far from completely understood, but suggests a constant competition for DSBs-mediated chromatin binding between 53BP1 and BRCA1-BARD1 complexes. Therefore, the common affinity of 53BP1 and BARD1 for H2AK15ub-modified nucleosomes, but their inverse affinities for disparate methylation states of H4K20, provides key evidence for the respective preferences of the proteins for pre- and post-replicative chromatin in a DSB-dependent manner, and for establishment of DSB repair pathway choice [16, 17, 61, 63, 65]. Likewise, this also underscores the potential contribution of SETD8 in choice of DSB repair pathway, especially correlates SETD8 oscillation to recruitment of 53BP1/BRCA1 complex in a cell cycle-dependent-specificity.

## The clinical impact of SETD8 deficiency

The proper regulation of chromatin organization guarantees genome integrity which is faithfully delivered to daughter cells during mitosis [18]. Persuasive evidence indicates that SETD8 exerts the biological functions through its interaction with a set of target nuclear proteins to ensure the genomic stability that are closely related to tissue development, senescence and tumorigenesis [36, 91, 92]. Experiments with gene-knockout mice showed that SETD8 is essential for mammalian development, since depletion of *Setd8* leads to pre-implantation and embryonic lethality [18, 93]. Consistently, SETD8 is involved in spermatogenesis via interaction with meiotic gatekeeper Stimulated By Retinoic Acid 8 (STRA8) [94]. Besides, SETD8 has also been implicated in carcinogenesis in various cancers, including prostate, breast, bladder, lung, papillary thyroid, pancreatic, hepatocellular carcinomas, and glioma [1, 4, 5, 20, 37, 82, 89, 95–97]. Most advanced cancer cells metastasize to other tissues of body and the epithelial-mesenchymal transition (EMT) is deemed as an initial step of cancer metastasis [4, 96, 98]. Several lines of evidence suggest that SETD8 is involved in the EMT [4, 20, 95, 96]. For example, evidence shows that SETD8 induces the EMT and enhances metastasis in prostate and breast cancers by cooperating with Zinc finger E-box-binding homeobox 1 (ZEB1), a transcriptional repressor, and the transcriptional factor TWIST, respectively [95, 96].

Cellular senescence is an irreversible growth arrest that contributes to development, anti-tumorigenesis and age-related disorders. It is verified that SETD8 deficiency alone is sufficient to elicit senescence, indicative of SETD8 as a barrier to prevent cellular senescence [36, 99]. Further findings substantiate that the repressive effect of SETD8 in senescence is achieved by maintaining the silencing mark H4K20me1 at the locus of the senescence switch gene p21, implying importance of methyltransferase activity of SETD8 [36]. Accordingly, another supportive study indicates that SETD8 suppresses nucleolar and mitochondrial activities to prevent cellular senescence through histone H4K20 mono-methylation [99]. Moreover, the ubiquitin-specific peptidase 17 like family member (USP17) is reported to prevent cellular senescence by removing ubiquitin marks from SETD8 so as to stabilize the methyltransferase, which confers the ability to promote production of H4K20me1 and transcriptionally repress p21 [91].

The cumulative findings therefore imply that SETD8 may have a potential to be a therapeutic target. Indeed, for example, recent work has shown that loss of BRCA1-associated protein 1 (BAP-1), a tumor repressor, results in a dramatic decrease in H4K20me1, catalyzed by SETD8, which sensitizes tumor cells to EZH2 pharmacologic inhibition and highlights a novel therapeutic approach for BAP1-mutant malignancies [100]. Collectively, SETD8 and its corresponding H4K20 methylation are closely related to tissue development, tumorigenesis and age-related disorders, which potentiates SETD8 as a potential therapeutic target to improve current human disease interventions [36, 91].

## Conclusions

In this review, we have discussed the roles for the methyltransferase SETD8 in DNA damage repair, especially involving the establishment of DSB repair pathway choice which is crucial for the timely elimination of DSBs, and underscored its therapeutic relevance. SETD8 is so far the only known lysine mono-methyltransferase in mammalian cells to produce H4K20me1, a prerequisite for di- and tri-methylation of H4K20, catalyzed by disparate methyltransferases [7, 19, 22]. Importantly, SETD8 is substantiated to be related to a number of cellular activities through histone H4 modification, including the DDR, which ultimately impinges upon tissue development, tumorigenesis and age-related disorders [1, 4, 18, 36, 91].

DSBs are cytotoxic with deleterious consequences if not repair properly. The HDR and c-NHEJ are two most prominent DSB repair pathways, the choice of which is mainly governed by the opposing activity of the two crucial proteins BRCA1 and 53BP1 [10–12, 15]. It is suggested that SETD8 and its corresponding H4K20 methylation are related to the recruitment of 53BP1 and BRCA1, thereby participating in DSBs repair [16, 17]. Recent evidence has revealed that 53BP1 recognizes H2AK15ub and H4K20me2 to promote c-NHEJ in G1 phase, whereas BRCA1-BARD1 complex binds to H2AK15ub and H4K20me0 to facilitate HR-directed DSB repair in S/G2 phase [17, 18, 53]. That is, chromatin engagement of 53BP1 and BRCA1 complex both requires bivalent interactions with histone H2A and H4 tails, through their recognition of shared H2AK15ub-modified nucleosomes, but distinct methylation states of H4K20, to ensure their respective preference for chromatins in a DSB-dependent-specify and a cell cycle-dependent-specify, respectively [17, 61, 63].

Clinically, previous studies have revealed that SETD8 is highly expressed in several types of cancers and an decrease in SETD8 expression is associated with a better survival rate, implying SETD8 as a potential therapeutic target in human disease interventions [1, 4, 5, 20, 37, 82, 89, 95–97]. Although the findings to date highly improved our understandings in roles for SETD8 and its corresponding H4K20 methylation in DSBs repair, in particular for the choice of DSB repair pathways, the cross-talk between HDR and c-NHEJ remains to be elucidated. Likewise, it also can be expected that more yet-to-be-identified methylation substrates of SETD8 may be uncovered. These future observations will not only expand the function of SETD8 and illuminate the molecular basis of its therapeutic relevance, but also further facilitate SETD8-H4K20 axis to be a viable therapeutic option for clinical applications.

## Data Availability

Not applicable.

## Abbreviations

HKMTs: Histone lysine methyltransferases
DSBs: DNA double-strand breaks
HDR: Homology-directed repair
c-NHEJ: Canonical nonhomologous end-joining
53BP1: TP53-binding protein 1
BRCA1: Breast cancer susceptibility protein-1
SET domain: Su(var)3-9, Enhancer of Zeste, Trithorax domain
MMSET: Multiple myeloma SET domain
WHSC1: Wolf-Hirschhorn syndrome candidate 1
PHF8: PHD finger protein 8
PHF2: PHD finger protein 2
DDR: DNA Damage Response
PCNA: Proliferating cell nuclear antigen
H4K16Ac: Acetylation of histone H4 on lysine 16
PTB domain: Phosphotyrosine-binding domain
SSA: Single-strand annealing
alt-EJ: Alternative end-joining
ssDNA: Single-stranded DNA
CtIP: CtBP-interacting protein
RIF1: Rap1-interacting factor 1
KAP-1: KRAB-associated protein 1
ATM: Ataxia-telangiectasia mutated kinase
ATR: Ataxia-telangiectasia Rad3‐related kinase
DNA-PK: DNA dependent protein kinase
PIKKs: Phosphatidylinositol 3-kinase-like protein kinase
RNF8: Ring finger protein 8
RNF168: Ring finger protein 168
Crb2: Crumbs cell polarity complex component 2
FFR: Central focus forming region
OD: Oligomerization domain
GAR: A glycine/arginine-rich motif
UDR: Ubiquitin-dependent recruitment motif
H2AK15ub: H2A ubiquitination at Lysine 15
L3MBTL1: Malignant-brain-tumor (MBT) protein
pMEFs: Primary mouse embryonic fibroblasts
FA: The Fanconi anemia
BARD1: BRCA1-associated RING domain protein 1
BRCA2: Breast cancer susceptibility gene 2
BRCT: BRCA1 C-terminal repeats
CC: Coiled-coil domain
RING: Really interesting new gene domain
ANK repeat: Ankyrin repeat
BUDR: BRCT domain ubiquitin-dependent recruitment motif
STRA8: Stimulated by retinoic acid 8
EMT: Epithelial-mesenchymal transition
ZEB1: Zinc finger E-box-binding homeobox 1
USP17: Ubiquitin-specific peptidase 17 like family member
BAP-1: BRCA1-associated protein 1
